# Association of outdoor artificial light at night with myopia among Chinese adolescents: a representative cross-sectional study

**DOI:** 10.3389/fmed.2024.1469422

**Published:** 2024-09-27

**Authors:** Ting Liu, Weixing Tan, Youjuan Fu, Beijing Cheng, Hua Tian, Can Liu, Zhixiang Wang, Yanting Zhang, Suzhen Guan, Zhihong Liu

**Affiliations:** ^1^School of Public Health, Ningxia Medical University, Yinchuan, China; ^2^Hospital Infection Management Department, People’s Hospital of Ningxia Hui Autonomous Region, Ningxia Medical University, Yinchuan, China; ^3^Key Laboratory of Environmental Factors and Chronic Disease Control, Yinchuan, China; ^4^School Health Section, Ningxia Center for Disease Control and Prevention, Yinchuan, China; ^5^School of Inspection, Ningxia Medical University, Yinchuan, China; ^6^Key Laboratory of Environmental Medicine Engineering, Ministry of Education, School of Public Health, Southeast University, Nanjing, China

**Keywords:** ALAN, artificial light at night, myopia, representative cross-sectional study, Chinese adolescents

## Abstract

**Background:**

The association between the rapid increase in myopia among adolescents and the amount of outdoor artificial light at night (ALAN) remains unclear. The aim of this study was to investigate the association between outdoor ALAN and myopia in adolescents.

**Methods:**

Stratified cluster random sampling was used to obtain a sample of 33,160 students (age range: 9–18 years; mean: 13.51 years) with complete data from 120 primary and secondary schools across the Ningxia region in China in 2021. Myopia was defined as a spherical equivalent (SE) ≤−0.5 diopters (D) in at least one eye, determined by automated refractometers without cycloplegia. Outdoor ALAN data were obtained from satellite data and the two-year average outdoor ALAN exposure for each participant was determined by matching it to their school address (home addresses were not available). The association between ALAN and myopia was assessed using multiple logistic regression models and restricted cubic spline (RCS) regression. Stratified analyses were performed by age, sex, residence, school level, and outdoor exercise time.

**Results:**

The myopia group had higher outdoor ALAN levels than the non-myopia group [median (interquartile spacing): 14.44 (3.88–26.56) vs. 6.95 (1.21–21.74) nanoWatts/cm^2^/sr]. After adjusting for covariates identified through stepwise regression, it was observed that the prevalence of myopia increased by 4% for every 10-unit change [95% confidence interval (CI): 1.02–1.07]. Compared to the first quantile (Q1) of outdoor ALAN exposure, the odds ratio (OR) of myopia was 1.20 (95% CI: 1.08–1.34) in the fourth quantile. RCS further showed a positive nonlinear relationship between outdoor ALAN exposure and myopia (*p* for nonlinear <0.001). Stronger effects were not found in subgroup analyses.

**Conclusion:**

Outdoor ALAN exposure is positively and nonlinearly associated with the prevalence of myopia in adolescents. Controlling outdoor light pollution may constitute a potential strategy to reduce the incidence of myopia in adolescents.

## 1 Introduction

Myopia is a refractive error in eye health, leading to blurred vision when observing distant objects (1–3). According to Holden et al. (4), the global prevalence of myopia in 2000 was 22.9% (1.406 billion people). Projections indicated that by 2050, myopia will affect 49.8% (4.758 billion) of the global population (4). Myopia occurs primarily in childhood through early adulthood, making adolescents a key group of concern (5, 6). Myopia poses a high risk for irreversible visual impairment caused by diseases such as glaucoma, retinal detachment, or myopic maculopathy. The increasing prevalence and associated risks not only escalate healthcare costs but also diminish the quality of life, posing a substantial burden on the global healthcare and economy (7–9). Consequently, a comprehensive understanding of the influencing factors of myopia becomes imperative.

Factors influencing myopia development include genetics, environment, and their interplay (10–12). Among these, inadequate sunlight exposure or limited time spent outdoors during daylight is usually suggested as an important driver of myopia prevalence (13, 14). However, outdoor artificial light at night (ALAN) differs from natural light during the day, potentially yielding distinct effects (14, 15). ALAN, with its high intensity, unnatural wavelengths, and abrupt switches, poses potential health risks (16, 17). Furthermore, in contemporary times, ALAN is growing at an annual rate of 5–20% (18), thus warranting our attention.

A review also highlighted the non-coincidental correlation between the widespread use of light-emitting diodes (LEDs) and the accelerating spike in the prevalence of myopia (14). Several studies have provided some plausible explanations for the above phenomenon (14, 19–22). Chronic exposure to ALAN may lead to severe circadian rhythm disturbances that alter the circadian rhythm of the retina (23, 24). Recent reviews have elucidated how retinal circadian rhythm disruption affects the onset and progression of myopia (14, 20, 21, 25). Additionally, ALAN suppressed melatonin production and deprived people of sleep, both of which were also important factors in the development of myopia (19, 22). Numerous epidemiological studies have investigated the association between ALAN and various outcomes, including obesity, fasting blood glucose, blood pressure, and sleep (18, 26–29). However, the association with myopia is notably limited.

A few epidemiologic studies have attempted to explore the relationship between nighttime light exposure and myopia development, but the results have been inconsistent (30–33). Quinn et al. (33) found that the development of myopia in children was strongly associated with their exposure to nighttime lighting in the first 2 years of life. However, subsequent research by Gwiazda et al. (32) found no significant relationship between myopia prevalence and exposure to nighttime lighting during the first 2 years or later. Similarly, Czepita et al. (30) reported that light from fluorescent lamps was associated with a higher incidence of astigmatism, but Czepita et al. (31) later found no association between nighttime light usage and myopia in Polish children without a family history of myopia. Additionally, to our knowledge, no available studies have explored the associations of ALAN with myopia among Chinese adolescents. To address the knowledge gap, we conducted a large representative cross-sectional study in 2021 among school adolescents in 22 districts/counties/cities in Ningxia, China. The study aimed to quantify the association between ALAN and myopia, and to examine the modifying effects of certain individual characteristics.

## 2 Methods

### 2.1 Study design and population

**Figure 1:**
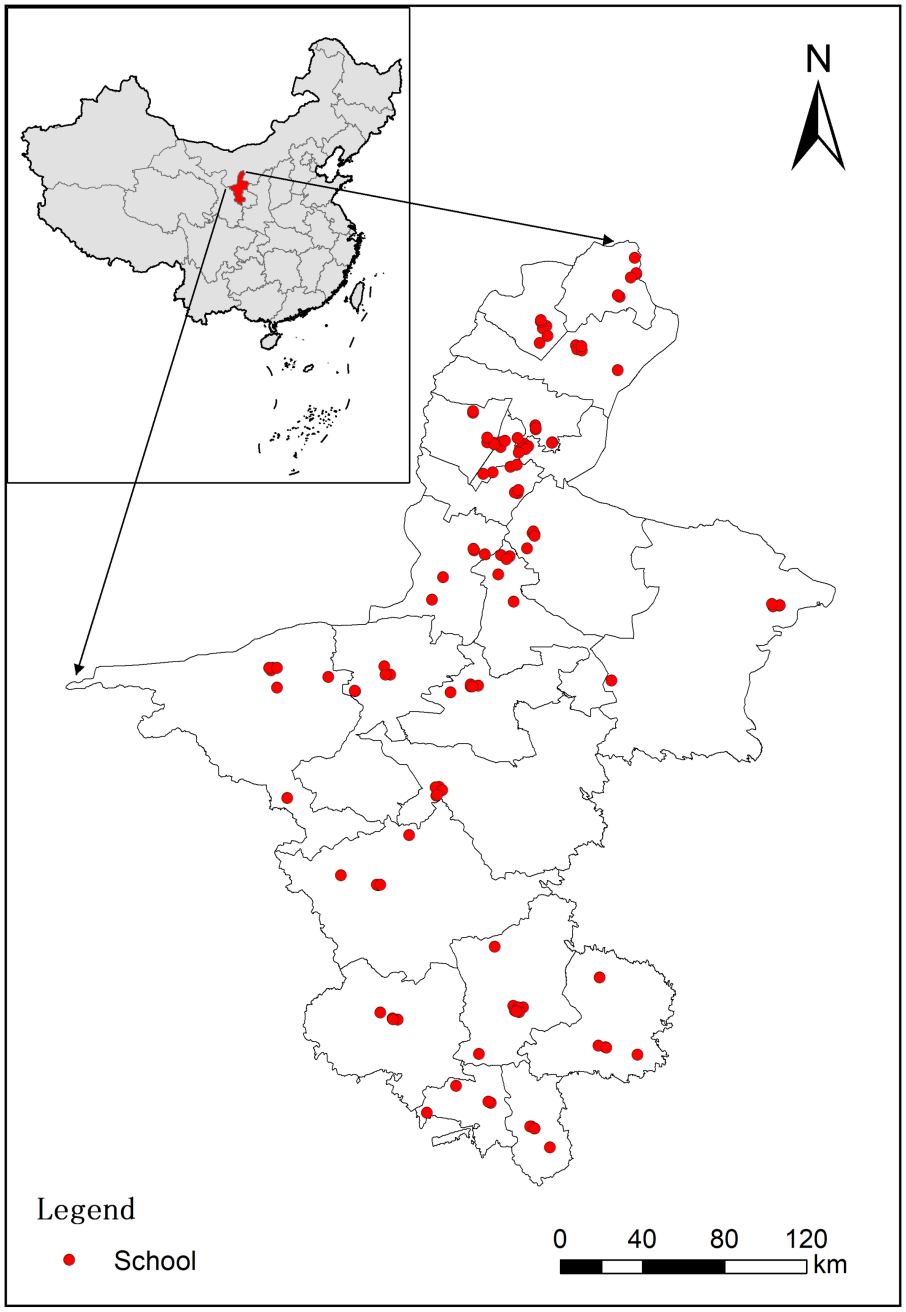
Spatial distribution of schools of the study subjects in Ningxia province, China. Red dots represent the schools (*n* = 120) of the study subjects; the gray lines represent administrative boundaries of 22 districts/counties/cities in Ningxia province, China.

The survey samples were obtained from the monitoring program of students’ myopia and health influencing factors conducted from September to December 2021 by the Ningxia Center for Disease Control and Prevention. Ningxia is located in the northwest of China, with 9 districts, 11 counties and 2 cities under its jurisdiction, covering a total area of 66,400 square kilometers.^1^ Ningxia is also an autonomous region of China known as the Ningxia Hui Autonomous Region. We used stratified cluster random sampling to select schools for monitoring, and a total of 120 schools were selected. The detailed spatial distribution of surveyed schools in each district or county is shown in Figure 1. About two classes per school per grade were taken as the basic unit of investigation. Given responsiveness, our study only focused on in-school adolescents (fourth grade through high school). A total of 34,777 adolescents participated in the survey, with each participant receiving a physical examination and a structured questionnaire. The questionnaires were paper-based and completed by the adolescents themselves, with completion taking approximately 10 to 30 min. After excluding 1,617 (4.65%) participants with missing covariates, a total of 33,160 adolescents were included in the final analysis. The study was approved by the Ethics Committee of the People’s Hospital of Ningxia Hui Autonomous Region ([2022]-NZR-093) and was conducted in accordance with the guidelines of the Declaration of Helsinki.

### 2.2 Myopia determination

In this study, myopia was diagnosed following the national standard “Technical Specifications for Student Health Examination” (GB/T 26343) and the health industry standard “Specification for Myopia Screening in Primary and Secondary School Students” (WS/T 663-2020). Briefly, ophthalmologists or trained investigators used automated refractometers to perform eye examinations without cycloplegia. All refractometers were compliant with the ISO 10342:2010 standard for ophthalmic instruments. Three measurements were taken in each eye and averaged. If the spherical equivalent (SE) of any two measurements differed by ≥0.50 diopters (D), the eye was re-measured and a new mean value was calculated. SE was calculated by the following formula: SE = sphericity degree + 0.5 × cylindrical degree. SE was used to assess refractive error in this study, consistent with previous epidemiologic surveys of children (34, 35). Based on previous literature and industry standards (WS/T 663-2020), myopia was defined as a SE of ≤−0.5 D in at least one eye (36, 37). In addition, students with suspected eye abnormalities were referred to a specialist for further evaluation.

### 2.3 Environmental data

During the survey, the residential address of each participant’s school was collected and subsequently the longitude and latitude of each participant’s school were extracted. Outdoor ALAN data were sourced from NASA/NOAA’s Visible Infrared Imaging Radiometer Suite (VIIRS) Day/Night Band (DNB) low-light imaging data, which provides high-quality nighttime light products^2^ (18, 38). The intensity of outdoor ALAN was quantified in raster units, ranging from 0 to 472.86 nanoWatts/cm^2^/sr, with a spatial resolution of 500 m. Taking Supplementary Figure S1 as an example, we presented the annual average outdoor ALAN exposure for 2020 in Ningxia province. Considering our population data collection occurred in 2021, we downloaded outdoor ALAN data for the years 2018–2020. Then, using ArcGIS version 10.2 software, we extracted the annual mean values of outdoor ALAN for each school buffer (1 km, 3 km, and 5 km) for the years 2018–2020. Considering that the majority of the included schools follow a three-year curriculum, for the main analysis, we used the 1 km buffer and 2-year average of outdoor ALAN exposure as the main exposure variable. Also, the effects of multiple definitions of outdoor ALAN were explored.

The assessment of the schools’ green space was conducted using the normalized difference vegetation index (NDVI). NDVI values range from −1 to 1 (3). Higher NDVI values indicate higher levels of green space around the school. NDVI data were sourced from Moderate Resolution Imaging Spectroradiometer (MODIS) surface reflectance data.^3^ Considering the seasonality of vegetation, we extracted the summer three-month average (June to August) from the MODIS VI product (MOD13Q1) as the primary exposure, with a spatial resolution of 250 m. Taking Supplementary Figure S2 as an example, we also presented the annual average NDVI exposure for 2020 in Ningxia province. For the main analysis, we also used the 1 km buffer and 2-year average of NDVI exposure as the main exposure variable.

The daily data of particulate matter ≤2.5 mm (PM_2.5_) at a 1 km spatial resolution were obtained from ChinaHighAirPollutants database^4^ (39). PM_2.5_ was ultimately selected as the primary exposure variable given the generally high correlation of air pollution indicators. Additionally, using ArcGIS version 10.2 software, the 1 km data were resampled to 500 m for subsequent matching purposes. For the main analysis, the 1 km buffer and 2-year average of PM_2.5_ exposure was used as the main exposure variable.

### 2.4 Covariates

Based on previous studies (3, 40, 41), covariates were selected as follows: demographic characteristics [age, sex, residence, nationality, school level, exposure time, body mass index (BMI), and parental myopia]; behavioral and lifestyle factors (nighttime sleep, outdoor exercise time per day, frequency of sugar per week, frequency of oil per week, frequency of fruit per week, frequency of vegetable per week, computers and TVs spend time per day, after-school homework time per day, after-school tutoring time per day, eye exercises per day, and annual frequency of visual inspections); and regional macro-indicators [gross domestic product (GDP) *per capita*, population density, and health technical personnel].

Among these factors, exposure time represented time exposed to the school environment: grade 4 to 6, indicating >3 year of exposure time; grade 7 and grade 10, indicating 1 year of exposure time; grade 8 and grade 11, indicating 2 year of exposure time; grade 9 and grade 12, indicating 3 year of exposure time, respectively. BMI was calculated by dividing the weight (kg) of each participant by the square of height (m). Data on demographic and behavioral/lifestyle factors were collected using a structured questionnaire. Behavioral/lifestyle factors were primarily assessed through questions about the past week. For example, participants were asked: “How much time is spent on outdoor exercise per day over the past week?” with response options including: less than 1 h, 1–2 h, and 2 h or more. Data on regional macro-indicators were obtained from Ningxia Statistical Yearbook of 2020.^5^

### 2.5 Statistical analysis

Continuous variables that were normal or approximately normal were described by the mean [standard deviation (SD)], while those that were not normally distributed were expressed as the median [interquartile spacing (IQR)]. Descriptive statistics for categorical variables were expressed as numbers (percentages, %). Depending on the distribution of the variables, student’s *t*-tests, Mann–Whitney *U*-tests, or chi-square tests were used to compare differences in individual characteristics between non-myopic and myopic participants.

Multiple logistic regression was used to estimate the associations between outdoor ALAN (per 10 units or per quartile increase) exposure and the prevalence of myopia. We constructed three models. Model 1 was not adjusted for any covariates. Based on univariate analysis (*p* < 0.1), Model 2 adjusted for age, sex, residence, nationality, school level, exposure time, body mass index, parental myopia, nighttime sleep, outdoor exercise time, fruit, vegetable, computers and TVs spend time, after-school homework time, after-school tutoring time, eye exercises, annual frequency of visual inspections, health technical personnel, NDVI, and PM_2.5_. A stepwise selection approach was used to build a parsimonious model. Thus, Model 3 (main model) adjusted for age, sex, residence, nationality, school level, exposure time, body mass index, parental myopia, fruit, vegetable, after-school homework time, eye exercises, annual frequency of visual inspections, and PM_2.5_.

In examining the relationship between outdoor ALAN exposure and the prevalence of myopia, we further investigated the shape of the exposure-response curve using restricted cubic spline (RCS) regression. For the RCS, the knots were set to the 5th, 25th, 50th, 75th and 95th percentiles, and the reference value was 0. The nonlinear *p*-value of the RCS was calculated by the Wald test.

Based on prior literature and our database, we selected five individual characteristics to explore the modifying effects, which were associated with myopia with moderate to high strength of evidence (3, 40). These characteristics were: (1) age (≤10 years and >10 years), (2) sex (male and female), (3) residence (rural areas and urban areas), (4) school level (primary, junior, and senior), and (5) outdoor exercise time (<1 h, 1–2 h, and ≥2 h). We used *Z*-tests to examine whether the differences between the subgroup effect estimates were significant or not. In addition, multiple comparisons existed for school level and outdoor exercise time. Thus, the Benjamini–Hochberg FDR method was calculated to correct for multiple comparisons.

We also performed a series of sensitivity analyses. First, we redefined four ALAN levels based on a variety of buffers (e.g., 3 km and 5 km) and annual averages (e.g., 1-year and 3-year average). Second, we excluded exposures with a duration of ≤1 year. Third, considering potential data clustering in schools, we used a generalized linear mixed model to test main results. All analyses were completed using SPSS for Windows (version 23.0) and R software (version 4.1.3). A two-tailed *p*-value of less than 0.05 was considered statistically significant.

## 3 Results

### 3.1 Descriptive statistics

Supplementary Table S1 shows the characteristics of 33,160 Chinese adolescents. The age of the students participating in the study ranged from 9 to 18 years (mean ± SD: 13.51 ± 2.55 years). Supplementary Table S1 also shows that the overall prevalence of myopia among students was 61.4% (20,325 out of 33,160). Table 1 further illustrates the variations in myopia prevalence across different groups. For example, a higher prevalence of myopia was observed among older adolescents, females, individuals from urban areas, and those of Han Chinese ethnicity, as well as among individuals with higher levels of education. Briefly, with the exception of five variables (outdoor exercise time, frequency of sugar, frequency of oil, GDP *per capita*, population density), all other variables showed differences between the two groups (*p* < 0.05).

**Table 1:** Description of non-myopia (*n* = 12,808) and myopia (*n* = 20,352) groups.

Variables^a^		Non-myopia [*N* (%)/mean ± SD/median (IQR)]	Myopia [*N* (%)/mean ± SD/median (IQR)]	*t*/*Z*/*χ*^2^	*p*-value
Age (years)		12.57 ± 2.31	14.10 ± 2.51	−57.08	<0.001
Sex	Male	7,270 (44.34)	9,127 (55.66)	446.50	<0.001
	Female	5,538 (33.04)	11,225 (66.96)		
Residence^b^	Rural areas	4,546 (52.39)	4,131 (47.61)	939.53	<0.001
	Urban areas	8,262 (33.75)	16,221 (66.25)		
Nationality^c^	Han nationality	5,907 (32.74)	12,134 (67.26)	577.64	<0.001
	Other nationalities	6,901 (45.64)	8,218 (54.36)		
School level^d^	Primary	6,656 (54.14)	5,637 (45.86)	2713.18	<0.001
	Junior	4,500 (37.24)	7,585 (62.76)		
	Senior	1,652 (18.81)	7,130 (81.19)		
Exposure time^e^	1 year	2,490 (35.80)	4,466 (64.20)	469.96	<0.001
	2 years	1,999 (28.85)	4,930 (71.15)		
	≥3 years	8,319 (43.16)	10,956 (56.84)		
Body mass index (kg/m^2^)^f^		19.99 ± 3.70	19.74 ± 3.64	5.85	<0.001
Parental myopia^g^	No	10,349 (43.15)	13,635 (56.85)	748.54	<0.001
	Yes	2,459 (26.80)	6,717 (73.20)		
Nighttime sleep (h)		8.51 ± 1.37	7.85 ± 1.47	41.29	<0.001
Outdoor exercise time per day	<1 h	2,610 (38.02)	4,255 (61.98)	4.71	0.095
	1–2 h	5,007 (38.23)	8,090 (61.77)		
	≥2 h	5,191 (39.33)	8,007 (60.67)		
Frequency of sugar per week	Never or sometimes	12,117 (38.63)	19,253 (61.37)	0.00	0.985
	Everyday	691 (38.60)	1,099 (61.40)		
Frequency of oil per week	Never or sometimes	12,317 (38.56)	19,627 (61.44)	1.64	0.201
	Everyday	491 (40.38)	725 (59.62)		
Frequency of fruit per week	Never or sometimes	4,214 (37.46)	7,036 (62.54)	9.78	0.002
	Everyday	8,594 (39.22)	13,316 (60.78)		
Frequency of vegetable per week	Never or sometimes	1,960 (42.57)	2,644 (57.43)	35.13	<0.001
	Everyday	10,848 (37.99)	17,708 (62.01)		
Computers and TVs spend time per day	<1 h	6,247 (40.36)	9,232 (59.64)	62.75	<0.001
	1–2 h	3,826 (38.77)	6,043 (61.23)		
	≥2 h	2,735 (35.01)	5,077 (64.99)		
After-school homework time per day	<1 h	6,234 (49.56)	6,345 (50.44)	1226.58	<0.001
	1–2 h	4,347 (36.02)	7,722 (63.98)		
	≥2 h	2,227 (26.16)	6,285 (73.84)		
After-school tutoring time per day	<1 h	10,608 (39.19)	16,457 (60.81)	54.91	<0.001
	1–2 h	1,372 (39.27)	2,122 (60.73)		
	≥2 h	828 (31.83)	1,773 (68.17)		
Eye exercises per day	No	418 (33.07)	846 (66.93)	29.56	<0.001
	1–2 times	11,164 (38.54)	17,804 (61.46)		
	>2 times	1,226 (41.87)	1,702 (58.13)		
Annual frequency of visual inspections	No	1,652 (43.23)	2,169 (56.77)	153.70	<0.001
	1–2 times	8,293 (36.39)	14,496 (63.61)		
	>2 times	2,863 (43.71)	3,687 (56.29)		
GDP *per capita* (Yuan)		43,675 (32,145, 54,575)	43,675 (31,649, 65,407)	−1.49	0.135
Population density (population/km^2^)		100.85 (68.73, 195.30)	113.80 (67.49, 212.61)	−0.89	0.371
Health technical personnel (per 1,000 population)		6.64 (5.20, 8.19)	6.64 (5.23, 8.19)	−4.24	<0.001
NDVI		0.46 ± 0.12	0.45 ± 0.12	8.34	<0.001
PM_2.5_ (μg/m^3^)		32.82 ± 3.82	32.49 ± 4.05	7.35	<0.001
Outdoor ALAN (nanoWatts/cm^2^/sr)		6.95 (1.21, 21.74)	14.44 (3.88, 26.56)	−23.70	<0.001

SD, standard deviation; IQR, interquartile range; GDP, gross domestic product; NDVI, normalized difference vegetation index; PM_2.5_, particulate matter ≤2.5 mm; ALAN, artificial light at night. Data on demographic and behavioral/lifestyle factors were collected using a structured questionnaire. Data on regional macro-indicators were obtained from Ningxia Statistical Yearbook of 2020.

a Data were complete and there was no missing data.

b Defined by whether the school was in a rural or urban area.

c Other nationalities were mainly Hui nationality (*n* = 14,884, 98.4%).

d School level included primary (grades 4 through 6), junior (grades 7 through 9), and senior (grades 10 through 12).

e Exposure time to the school environment: grade 4 to 6, indicating >3 year of exposure time; grade 7 and grade 10, indicating 1 year of exposure time; grade 8 and grade 11, indicating 2 year of exposure time; grade 9 and grade 12, indicating 3 year of exposure time, respectively.

f BMI was calculated by dividing the weight (kg) of each participant by the square of height (m).

g Defined as one or both parents suffering from myopia.

The radiance of LAN exposure exhibited significant variation across Ningxia Province, as illustrated in Supplementary Figure S1. In general, most areas experienced lower-intensity outdoor ALAN, while higher-intensity outdoor ALAN was notably concentrated in northern cities. The distribution of outdoor ALAN among the 120 study sites (schools) displayed a leftward skew, and the median (IQR) ALAN exposure for all study sites was 12.90 (2.10, 25.04) nanoWatts/cm^2^/sr, as depicted in Supplementary Figure S3.

### 3.2 Associations of outdoor ALAN with myopia

**Figure 2:**
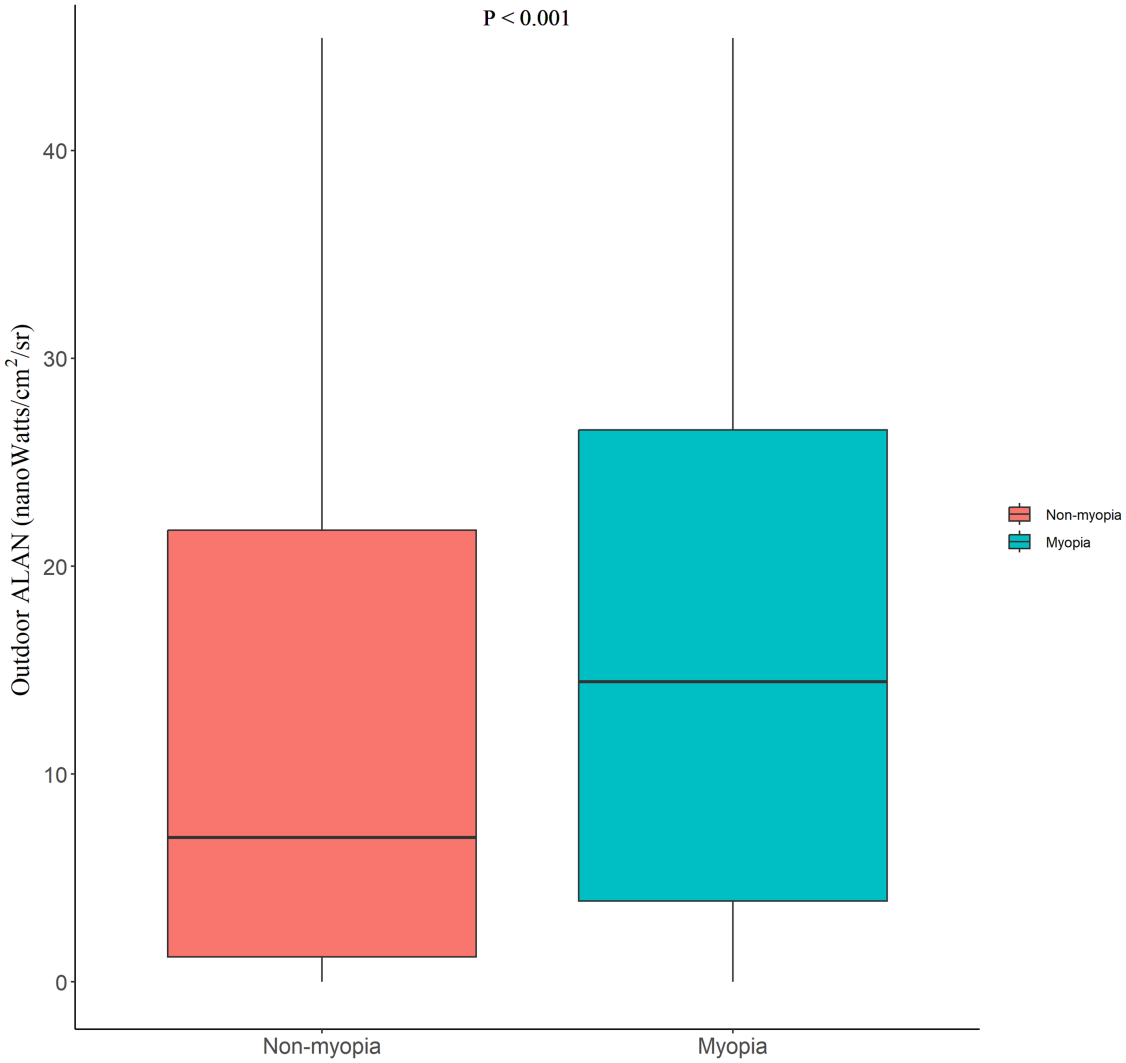
Differences in the outdoor ALAN between the non-myopia and myopia groups. “*p* < 0.001” represented a significant difference in the median by the Mann–Whitney *U*-test. ALAN, artificial light at night.

Significant differences in outdoor ALAN levels were observed between the non-myopia group and the myopia group (*p* < 0.001; see Figure 2). Outdoor ALAN levels were higher in the myopia group [median (IQR): 14.44 nanoWatts/cm^2^/sr (3.88–26.56)] than in the non-myopia group [median (IQR): 6.95 nanoWatts/cm^2^/sr (1.21–21.74)].

Table 2 presents the ORs and 95% CIs for the associations between outdoor ALAN exposure and the prevalence of myopia. In Model 1, without adjusting for any covariates, there was an 18% increase in myopia prevalence for every 10-unit change in outdoor ALAN. In Model 2, after adjusting for covariates identified through univariate analysis (*p* < 0.1), a 4% increase in myopia prevalence was observed for every 10-unit change in outdoor ALAN. Similar results were obtained using stepwise regression approaches in Model 3.

**Table 2:** Associations of outdoor ALAN with myopia in different models.

Models	OR (95% CI)	*p*-value
Model 1		
Continuous variable (per 10 units)	**1.18 (1.16, 1.20)**	**<0.001**
Quartile variable		
Q1	Reference	
Q2	**1.62 (1.52, 1.72)**	**<0.001**
Q3	**1.97 (1.85, 2.09)**	**<0.001**
Q4	**2.08 (1.95, 2.22)**	**<0.001**
Model 2		
Continuous variable (per 10 units)	**1.04 (1.01, 1.07)**	**0.002**
Quartile variable		
Q1	Reference	
Q2	1.07 (0.98, 1.18)	0.147
Q3	1.06 (0.95, 1.18)	0.309
Q4	**1.21 (1.08, 1.35)**	**0.001**
Model 3		
Continuous variable (per 10 units)	**1.04 (1.02, 1.07)**	**0.001**
Quartile variable		
Q1	Reference	
Q2	1.06 (0.97, 1.16)	0.190
Q3	1.04 (0.94, 1.16)	0.409
Q4	**1.20 (1.08, 1.34)**	**0.001**

ALAN, artificial light at night; OR, odds ratio; 95% CI, 95% confidence interval. Model 1: crude model. Model 2 (based on univariate analysis: *p* < 0.1): adjusted for age, sex, residence, nationality, school level, exposure time, body mass index, parental myopia, nighttime sleep, outdoor exercise time, fruit, vegetable, computers and TVs spend time, after-school homework time, after-school tutoring time, eye exercises, annual frequency of visual inspections, health technical personnel, NDVI, and PM_2.5_. Model 3 (based on stepwise selection approaches): adjusted for age, sex, residence, nationality, school level, exposure time, body mass index, parental myopia, fruit, vegetable, after-school homework time, eye exercises, annual frequency of visual inspections, and PM_2.5_. Statistical significance was indicated by bold font.

To preliminarily explore non-linear relationships, we categorized outdoor ALAN into quartiles (Q1, Q2, Q3, and Q4). As shown in Table 2, association estimates were calculated for the top three quartiles compared to the lowest outdoor ALAN quartile. In Model 1, statistically significant positive associations were observed between outdoor ALAN and myopia prevalence (OR_Q4 vs. Q1_: 2.08, 95% CI: 1.95–2.22). Model 2 and Model 3 found similar association estimates (OR_Q4 vs. Q1_: 1.21, 95% CI: 1.08–1.35; OR_Q4 vs. Q1_: 1.20, 95% CI: 1.08–1.34, respectively). Considering the simplicity of the model, we use Model 3 as the main model for subsequent analysis.

**Figure 3:**
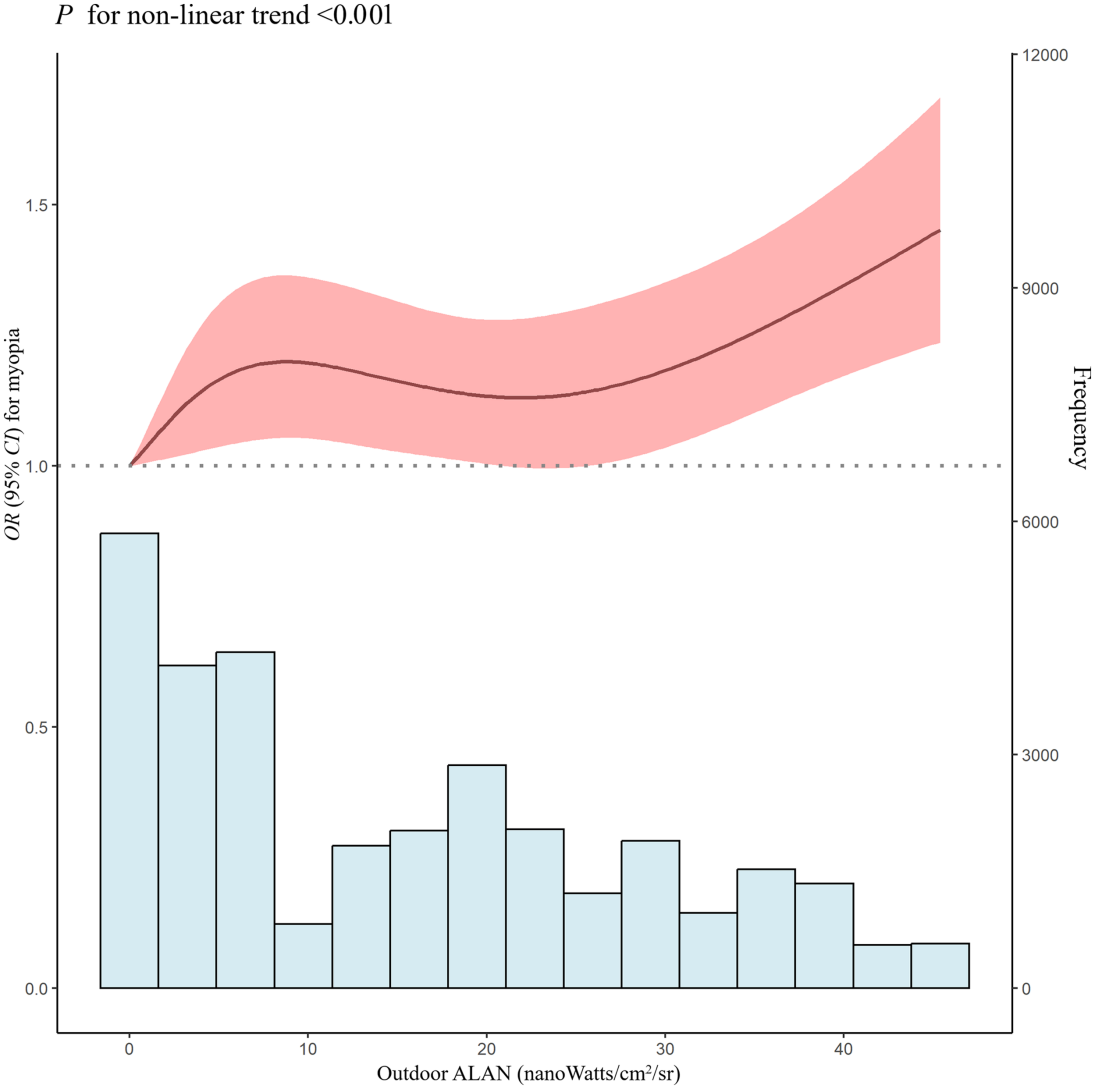
Nonlinear association of outdoor ALAN exposure with OR for myopia. ALAN, artificial light at night; OR, odds ratio; 95% CI, 95% confidence interval. Adjusted for age, sex, residence, nationality, school level, exposure time, body mass index, parental myopia, fruit, vegetable, after-school homework time, eye exercises, annual frequency of visual inspections, and PM_2.5_.

We further investigated the shape of the exposure-response curve in examining the relationship between outdoor ALAN exposure and myopia prevalence (Figure 3). The relationship between outdoor LAN exposure (reference concentration: 0 nanoWatts/cm^2^/sr) and the prevalence of myopia exhibited a nonlinear exposure-response curve (*p* for nonlinear <0.001). The effect steeply increased at low outdoor ALAN levels, remained stable at medium levels, and gradually increased at high levels. Overall, the RCS analysis indicated a nonlinear positive dose-response association between long-term outdoor ALAN exposure and myopia in adolescents.

### 3.3 Subgroup and sensitivity analysis

Table 3 shows the associations of outdoor ALAN with myopia prevalence modified by age, sex, residence, parental myopia, school level, and outdoor exercise time. Compared to the outdoor ALAN in Q1, the outdoor ALAN in Q4 was significantly associated with increased odds of myopia in adolescents older than 10 years of age, with no myopic parents, attending junior school, and spending more time outdoors. Both males and females showed a significant increase in the prevalence of myopia when exposed to outdoor ALAN in Q4 compared to Q1. Compared to the outdoor ALAN in Q1, the outdoor ALAN in Q2 was significantly associated with increased odds of myopia in rural areas. It is worth noting that we only found statistically significant differences between urban and rural areas (*p* = 0.029).

**Table 3:** Associations of outdoor ALAN with myopia by subgroups.

Quartile variable	Age			Quartile variable	School level^a^		
	≤10 years	>10 years	*p*_interaction_		Primary	Junior	*p*_interaction_
Q1	Reference	Reference		Q1	Reference	Reference	
Q2	1.06 (0.76, 1.47)	1.08 (0.98, 1.19)	0.907	Q2	1.08 (0.88, 1.32)	1.12 (0.99, 1.26)	0.918
Q3	1.38 (0.95, 2.02)	1.01 (0.90, 1.12)	0.273	Q3	1.21 (0.96, 1.52)	1.02 (0.89, 1.18)	0.690
Q4	1.37 (0.93, 2.00)	1.20 (1.07, 1.35)	0.529	Q4	1.14 (0.90, 1.44)	1.40 (1.20, 1.64)	0.801

Quartile variable	Sex			Quartile variable	School level^a^		
	Male	Female	*p*_interaction_		Primary	Senior	*p*_interaction_
Q1	Reference	Reference		Q1	Reference	Reference	
Q2	1.03 (0.91, 1.16)	1.10 (0.96, 1.26)	0.451	Q2	1.08 (0.88, 1.32)	0.89 (0.63, 1.26)	0.694
Q3	1.03 (0.90, 1.18)	1.06 (0.91, 1.23)	0.799	Q3	1.21 (0.96, 1.52)	0.81 (0.58, 1.13)	0.336
Q4	1.23 (1.07, 1.42)	1.18 (1.01, 1.38)	0.700	Q4	1.14 (0.90, 1.44)	1.12 (0.79, 1.58)	0.934

Quartile variable	Residence			Quartile variable	Outdoor exercise time per day^a^		
	Rural areas	Urban areas	*p*_interaction_		<1 h	1–2 h	*p*_interaction_
Q1	Reference	Reference		Q1	Reference	Reference	
Q2	1.13 (1.00, 1.30)	0.91 (0.79, 1.06)	**0.029**	Q2	0.92 (0.75, 1.15)	1.11 (0.97, 1.28)	0.557
Q3	1.04 (0.81, 1.35)	0.91 (0.78, 1.05)	0.945	Q3	0.86 (0.68, 1.10)	1.16 (0.99, 1.36)	0.282
Q4	—	1.05 (0.91, 1.22)	—	Q4	1.08 (0.84, 1.39)	1.28 (1.08, 1.51)	0.426

Quartile variable	Parental myopia			Quartile variable	Outdoor exercise time per day^a^		
	No	Yes	*p*_interaction_		<1 h	≥2 h	*p*_interaction_
Q1	Reference	Reference		Q1	Reference	Reference	
Q2	1.11 (1.00, 1.23)	0.90 (0.74, 1.10)	0.065	Q2	0.92 (0.75, 1.15)	1.07 (0.92, 1.23)	0.426
Q3	1.04 (0.92, 1.16)	1.01 (0.81, 1.26)	0.824	Q3	0.86 (0.68, 1.10)	1.02 (0.87, 1.19)	0.426
Q4	1.26 (1.12, 1.42)	1.03 (0.82, 1.29)	0.483	Q4	1.08 (0.84, 1.39)	1.20 (1.01, 1.41)	0.557

ALAN, artificial light at night; —, not analysis. Adjusted for age, sex, residence, nationality, school level, exposure time, body mass index, parental myopia, fruit, vegetable, after-school homework time, eye exercises, annual frequency of visual inspections, and PM_2.5_. Statistical significance between groups was indicated by bold font.

a The Benjamini–Hochberg FDR method was calculated to correct for multiple comparisons.

In sensitivity analyses, the associations between outdoor ALAN and myopia prevalence remained stable (see Supplementary Tables S2, S3). First, defining four ALAN levels was based on a variety of buffers (e.g., 3 km and 5 km) and annual averages (e.g., 1-year and 3-year average). The associations varied to some extent in different definitions, but the direction remained stable. Second, excluding exposure time ≤1 year, we observed a slight decrease in the effects (OR_Q4 vs. Q1_: 1.16, 95% CI: 1.02–1.31). Third, considering potential data clustering in schools, we used a generalized linear mixed model and observed the increased effects. For instance, a 8% increase in myopia prevalence was observed for every 10-unit change in outdoor ALAN. Compared to the outdoor ALAN in Q1, the outdoor ALAN in Q4 was significantly associated with increased odds of myopia (OR_Q4 vs. Q1_: 1.40, 95% CI: 1.00–1.96).

## 4 Discussion

### 4.1 Main findings

This study is the first to identify a positive nonlinear association between long-term exposure to outdoor ALAN and myopia in adolescents. Although a stronger correlation was not found in a specific population (e.g., sex), the findings still offer important insights. Given the rising rates of ALAN and myopia in contemporary society, the study offers crucial first-hand evidence of ALAN’s potential impact on eye health.

### 4.2 Comparison with previous work and possible explanations

Holden et al. (4) reported a global myopia prevalence of 22.9% (1.406 billion people) in 2000, projecting a significant increase to 49.8% (4.758 billion) by 2050 (4). A comprehensive review underscored a non-coincidental correlation between the widespread adoption of LEDs and the escalating prevalence of myopia (14). While daytime LEDs used for general lighting may impact visual performance and eye strain, ALAN, characterized by exposure to artificial light during nighttime hours, may have distinct and potentially more significant effects on myopia development (14, 25). Research on the association of ALAN with myopia is very limited, as only a few epidemiologic studies conducted abroad have shown inconsistent results (30–33). Quinn et al. (33) identified a strong link between childhood myopia development and exposure to nighttime lighting during the first 2 years of life. However, a follow-up study by Gwiazda et al. (32) found no significant relationship between nighttime lighting exposure—whether in the first 2 years or later—and the prevalence of myopia. Similarly, Czepita et al. (30) observed a high incidence of astigmatism associated with fluorescent lighting. In a subsequent study, Czepita et al. (31) found no association between myopia and nighttime light use in Polish children without a family history of myopia. These inconsistent results may partly be attributed to the accuracy of exposure measurements, statistical methods, and the limitation on specific populations.

Our study extends previous work in three ways. First, unlike previous studies that used questionnaires to assess artificial light (30–33), we used relatively high-precision data of outdoor ALAN (38). By focusing on outdoor ALAN, our study introduces a new perspective on environmental influences on myopia, offering insights beyond the commonly studied indoor lighting. Second, we used a variety of methods, including logistic regression and RCS methods, to mutually validate the results. These methods demonstrated a positive nonlinear dose–response relationship between ALAN and myopia, providing a more comprehensive understanding of the association. Third, to our knowledge, this is the first and largest study examining the link between ALAN and myopia in adolescent. These three extensions further add support for a positive association between ALAN and myopia.

The exact mechanisms between ALAN and myopia are not clear. One possible mechanism is that long-term exposure to ALAN may lead to severe disruption of circadian rhythms, thereby altering the circadian rhythm of the retina. Several systematic reviews have detailed this mechanism, including Lei et al. (27), Regmi et al. (42), and Nickla (25). In addition, several other systematic reviews have noted that retinal circadian rhythm disturbances may specifically affect the onset and progression of myopia, such as Chakraborty et al. (20), Stone et al. (21), and Zhang et al. (14). Another possible mechanism is that ALAN inhibits melatonin production and deprives sleep, both of which are also important factors in the development of myopia, as supported by Chakraborty et al. (19) and Touitou et al. (22). These mechanisms form, in part, the biological basis for the link between ALAN and myopia.

### 4.3 Public health and clinical implications

Our study found that long-term exposure to outdoor ALAN may be positively associated with myopia. This association provides important insights for clinical practice and public health. Ophthalmologists can advise adolescents to take a series of measures, such as avoiding overexposure to strong light sources at night, which are expected to reduce the incidence of myopia. Eye care professionals can advise adolescents to avoid or minimize exposure to ALAN, which may help reduce the incidence of myopia. For high-risk groups in light-polluted environments, especially students, regular eye health checkups are especially important, and early detection and intervention will improve the effectiveness of myopia prevention and control.

In addition, understanding the association between outdoor light pollution and myopia provides an important knowledge base for the public. Although no specific sensitive groups were identified, the effect did diminish after adjusting for other known risk factors such as lifestyle factors. This suggests that individuals may be able to take steps to mitigate the effects of ALAN. Educational institutions and health departments can work together to conduct publicity campaigns to raise public awareness of outdoor light pollution in order to reduce its harmful effects on eye health. It is crucial to incorporate light pollution prevention and control measures in urban planning, such as rationalizing lighting facilities and reducing glare exposure, to improve the urban environment and reduce eye health risks. Taken together, this study provides substantial guidance for the development of more effective eye health strategies that not only contribute to the eye health of individuals, but also have a positive impact at the public health level.

## 5 Strengths and limitations

Our study has several strengths. First, this is the first and largest research to explore the association between outdoor ALAN and adolescent myopia, using a representative sample. This makes our results more generalizable to adolescents in other parts of China, especially to populations in cities in northwestern China. Second, the study incorporated covariates of multiple dimensions (such as demographic characteristics, behavioral/lifestyle factors, and regional macro-indicators) to make the results closer to the real situation by adjusting for confounders as much as possible. Third, we conducted multiple sensitivity analyses and subgroup analyses to emphasize the robustness of our results.

Nevertheless, there are also several limitations. First, cross-sectional studies have inherent limitations and fail to provide evidence of a causal relationship between ALAN and myopia. Further cohort and experimental studies (including clinical trials and animal studies) are needed to elucidate these findings and to further our understanding of the mechanisms by which ALAN causes myopia in adolescents. Second, the outdoor ALAN data include only daily, monthly and annual data, not hourly data (see text footnote 2). Therefore, we were unable to assess the effects of ALAN exposure during the last few hours of the day on myopia. Future studies should develop and use hourly ALAN data to further validate and extend the current findings. Third, this study was only able to use ALAN data obtained from satellite remote sensing to represent ALAN exposure at participants’ schools. Individual participant addresses were not obtained, which may have led to measurement bias. Given that most schools in China use the principle of proximity to school, the ALAN values near schools reflect to some extent the level of residential exposure. It is undeniable that this measurement bias may underestimate the true effect of ALAN exposure. In future studies, we will collect individual addresses to better match exposure levels and reduce the likelihood of misclassification. Fourth, indoor ALAN exposure could not be measured. Indoor ALAN exposure might significantly impact myopia and potentially confound the results related to outdoor ALAN exposure (43). Thus, we measured alternative indicators (such as after-school homework time and after-school tutoring time) and adjusted for these variables to mitigate the potential impact of indoor ALAN exposure on the study results. Future studies should include both indoor and outdoor ALAN exposure to provide a more comprehensive understanding of how different types of light exposure affect myopia. Fifth, considering that urbanization may be an important confounder (3), we initially included three regional macro-indicators (GDP *per capita*, population density, and health technical personnel) and residence for potential adjustment in our analysis. Although some factors may not be included in the final model, they were carefully considered during the analysis. Finally, unmeasured variables may cause residual confounding (44), such as outdoor time during daylight. However, our model was adjusted for a range of important/alternative covariates (e.g., outdoor exercise time) that were closely associated with the outcomes. Therefore, we believe that the effects of unmeasured residual confounding on the results are likely to be limited and are unlikely to change the direction of the main results.

## 6 Conclusion

In conclusion, our study reveals a positive nonlinear relationship between long-term outdoor ALAN exposure and adolescent myopia. No stronger correlations were found in specific populations. These findings deepen our understanding of environmental influences on myopia. Further cohort studies are essential to clarify the association between outdoor ALAN and myopia, as well as to understand the underlying mechanisms that contribute to adolescent myopia.

## Data Availability

The data analyzed in this study is subject to the following licenses/restrictions: the complete data used throughout the study are available from the corresponding author upon reasonable request. Requests to access these datasets should be directed to ZL, lzh_2580@sina.com.

## Footnotes

^1^ http://nxdata.com.cn/

^2^ https://eogdata.mines.edu/products/vnl/

^3^ https://ladsweb.modaps.eosdis.nasa.gov/

^4^ https://weijing-rs.github.io/product.html

^5^ http://nxdata.com.cn/files_nx_pub/html/tjnj/2020/indexfiles/indexch.htm
