# Supplementary material for: Association of outdoor artificial light at night with myopia among Chinese adolescents: a representative cross-sectional study
Source: Front Med (Lausanne). 2024 Sep 27;11:1469422. doi: 10.3389/fmed.2024.1469422 (PMC11466777; doi:10.3389/fmed.2024.1469422)
Supplement: Supplementary file 1 [file Data_Sheet_1.docx]

**Supplementary materials**

**Table S1**

Characteristics of the 33,160 Chinese adolescents.

**Table S2**

Sensitivity analysis of associations of outdoor ALAN with myopia.

**Table S3**

Associations of outdoor ALAN with myopia using generalized linear mixed models.

**Fig. S1**

Average exposure of outdoor artificial light at night in Ningxia Province in 2020.

**Fig. S2**

Average exposure of normalized difference vegetation index in Ningxia Province in 2020.

**Fig. S3**

Histogram of outdoor artificial light at night in this study (n = 33,160).

**Table S1**

Characteristics of the 33,160 Chinese adolescents.

| Variables ^a^ |  | [N (%)/Mean ± SD/  Median (IQR)] |
| --- | --- | --- |
| Age (years) |  | 13.51 ± 2.55 |
| Sex | Male | 16397 (49.45) |
|  | Female | 16763 (50.55) |
| Residence ^b^ | Rural areas | 8677 (26.17) |
|  | Urban areas | 24483 (73.83) |
| Nationality ^c^ | Han nationality | 18041 (54.41) |
|  | Other nationalities | 15119 (45.59) |
| School level ^d^ | Primary | 12293 (37.07) |
|  | Junior | 12085 (36.44) |
|  | Senior | 8782 (26.48) |
| Exposure time ^e^ | 1 year | 6956 (20.98) |
|  | 2 years | 6929 (20.90) |
|  | ≥3 years | 19275 (58.13) |
| Body mass index (kg/m^2^) ^f^ |  | 19.84 ± 3.66 |
| Myopia | No | 12808 (38.6) |
|  | Yes | 20325 (61.4) |
| Parental myopia ^g^ | No | 23984 (72.33) |
|  | Yes | 9176 (27.67) |
| Nighttime sleep (h) |  | 8.11 ± 1.47 |
| Outdoor exercise time per day | <1 h | 6865 (20.70) |
|  | 1~2 h | 13097 (39.50) |
|  | ≥2 h | 13198 (39.80) |
| Frequency of sugar per week | Never or sometimes | 31370 (94.60) |
|  | Everyday | 1790 (5.40) |
| Frequency of oil per week | Never or sometimes | 31944 (96.33) |
|  | Everyday | 1216 (3.67) |
| Frequency of fruit per week | Never or sometimes | 11250 (33.93) |
|  | Everyday | 21910 (66.07) |
| Frequency of vegetable per week | Never or sometimes | 4604 (13.88) |
|  | Everyday | 28556 (86.12) |
| Computers and TVs spend time per day | <1 h | 15479 (46.68) |
|  | 1~2 h | 9869 (29.76) |
|  | ≥2 h | 7812 (23.56) |
| After-school homework time per day | <1 h | 12579 (37.93) |
|  | 1~2 h | 12069 (36.40) |
|  | ≥2 h | 8512 (25.67) |
| After-school tutoring time per day | <1 h | 27065 (81.62) |
|  | 1~2 h | 3494 (10.54) |
|  | ≥2 h | 2601 (7.84) |
| Eye exercises per day | No | 1264 (3.81) |
|  | 1~2 times | 28968 (87.36) |
|  | >2 times | 2928 (8.83) |
| Annual frequency of visual inspections | No | 3821 (11.52) |
|  | 1~2 times | 22789 (68.72) |
|  | >2 times | 6550 (19.75) |
| GDP per capita (Yuan) |  | 43675 (31649, 65407) |
| Population density (population/km^2^) |  | 100.85 (68.73, 212.61) |
| Health technical personnel (per 1,000 population) | | 6.64 (5.23, 8.19) |
| NDVI |  | 0.46 ± 0.12 |
| PM_2.5_ (μg/m^3^) |  | 32.62 ± 3.96 |
| Outdoor ALAN (nanoWatts/cm^2^/sr) |  | 12.90 (2.10, 25.04) |

Abbreviations: SD: standard deviation; IQR: interquartile range; GDP: gross domestic product; NDVI: normalized difference vegetation index; PM_2.5_: particulate matter ≤2.5 mm; ALAN: artificial light at night. Data on demographic and behavioral/lifestyle factors were collected using a structured questionnaire. Data on regional macro-indicators were obtained from Ningxia Statistical Yearbook of 2020.

^a^ Data were complete and there was no missing data.

^b^ Defined by whether the school was in a rural or urban area.

^c^ Other nationalities were mainly Hui nationality (n=14884, 98.4%).

^d^ School level included primary (grades 4 through 6), junior (grades 7 through 9), and senior (grades 10 through 12).

^e^ Exposure time to the school environment: grade 4 to 6, indicating >3 year of exposure time; grade 7 and grade 10, indicating 1 year of exposure time; grade 8 and grade 11, indicating 2 year of exposure time; grade 9 and grade 12, indicating 3 year of exposure time, respectively.

^f^ BMI was calculated by dividing the weight (kg) of each participant by the square of height (m).

^g^ Defined as one or both parents suffering from myopia.

**Table S2**

Sensitivity analysis of associations of outdoor ALAN with myopia.

| Models |  | OR (95% CI) | *P* value |
| --- | --- | --- | --- |
| 1 km buffer and 1-year average | Q1 | Reference |  |
|  | Q2 | 1.06 (0.97, 1.16) | 0.193 |
|  | Q3 | 1.05 (0.94, 1.16) | 0.381 |
|  | Q4 | **1.19 (1.07, 1.33)** | **0.001** |
| 1 km buffer and 3-year average | Q1 | Reference |  |
|  | Q2 | 1.05 (0.96, 1.15) | 0.280 |
|  | Q3 | 1.07 (0.96, 1.18) | 0.214 |
|  | Q4 | **1.22 (1.09, 1.35)** | **<0.001** |
| 3 km buffer and 2-year average | Q1 | Reference |  |
|  | Q2 | 1.05 (0.94, 1.17) | 0.371 |
|  | Q3 | 0.99 (0.90, 1.09) | 0.831 |
|  | Q4 | **1.23 (1.11, 1.36)** | **<0.001** |
| 5 km buffer and 2-year average | Q1 | Reference |  |
|  | Q2 | 1.06 (0.98, 1.15) | 0.121 |
|  | Q3 | 0.90 (0.81, 1.00) | 0.056 |
|  | Q4 | 1.09 (1.00, 1.19) | 0.063 |
| Exposure time ≥2 years | Q1 | Reference |  |
|  | Q2 | 1.02 (0.92, 1.14) | 0.649 |
|  | Q3 | 1.02 (0.91, 1.15) | 0.718 |
|  | Q4 | **1.16 (1.02, 1.31)** | **0.023** |

Abbreviations: ALAN: artificial light at night. Adjusted for age, sex, residence, nationality, school level, exposure time, body mass index, parental myopia, fruit, vegetable, after-school homework time, eye exercises, annual frequency of visual inspections, and PM_2.5_.

**Table S3**

Associations of outdoor ALAN with myopia using generalized linear mixed models.

| Models | OR (95% CI) | *P* value |
| --- | --- | --- |
| Continuous variable (per 10 units) | **1.08 (1.01, 1.16)** | **0.045** |
| Quartile variable | |  |
| Q1 | Reference |  |
| Q2 | 1.13 (0.84, 1.53) | 0.427 |
| Q3 | 1.09 (0.77, 1.54) | 0.618 |
| Q4 | **1.40 (1.00, 1.96)** | **0.043** |

Abbreviations: ALAN: artificial light at night. Adjusted for age, sex, residence, nationality, school level, exposure time, body mass index, parental myopia, fruit, vegetable, after-school homework time, eye exercises, annual frequency of visual inspections, and PM_2.5_.


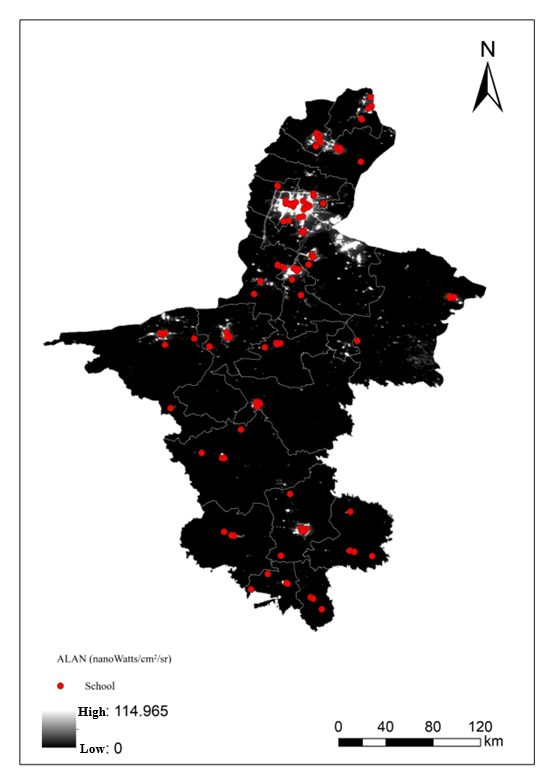
**Fig. S1**

Average exposure of outdoor artificial light at night in Ningxia Province in 2020.


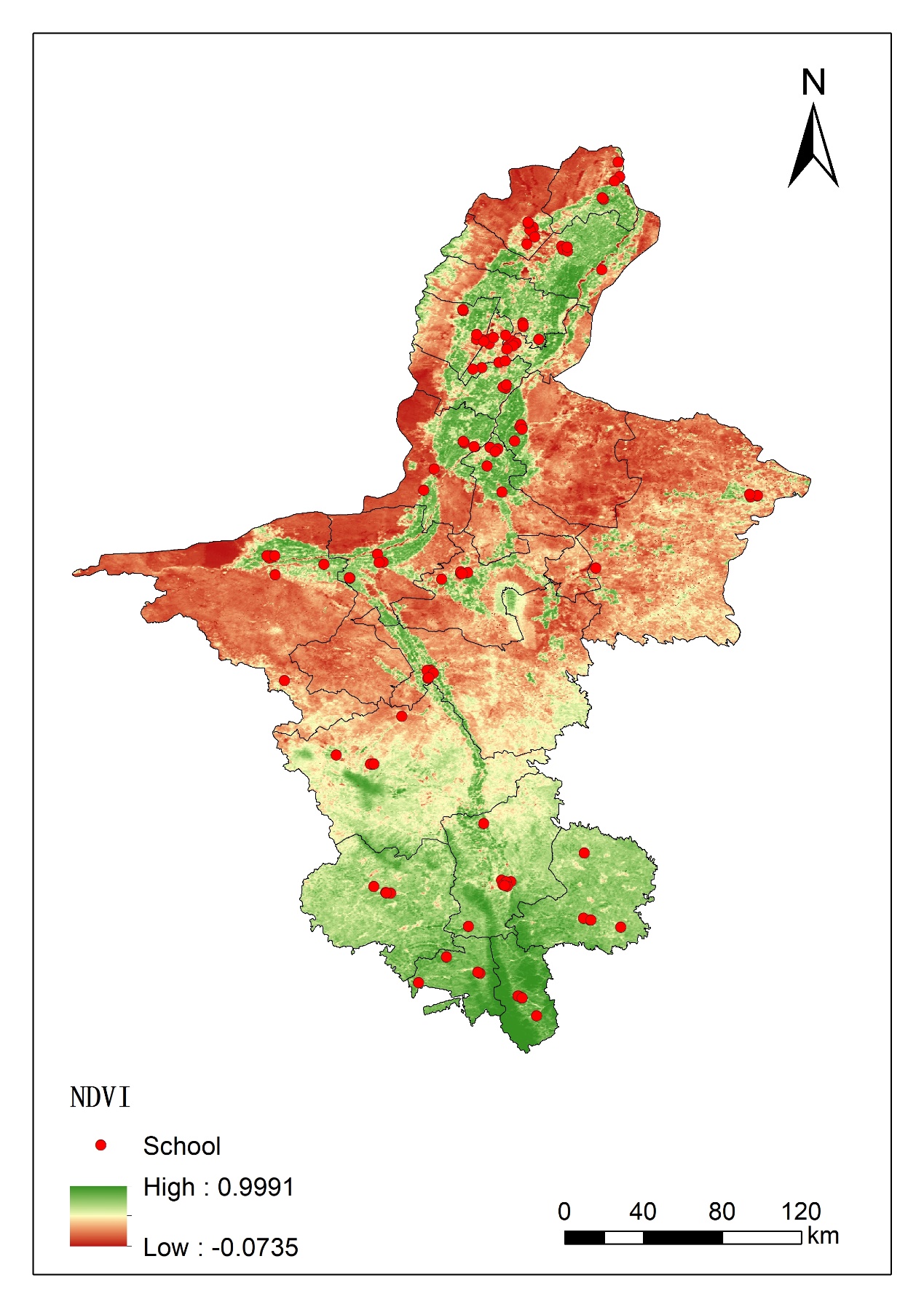
 **Fig. S2**

Average exposure of normalized difference vegetation index in Ningxia Province in 2020.


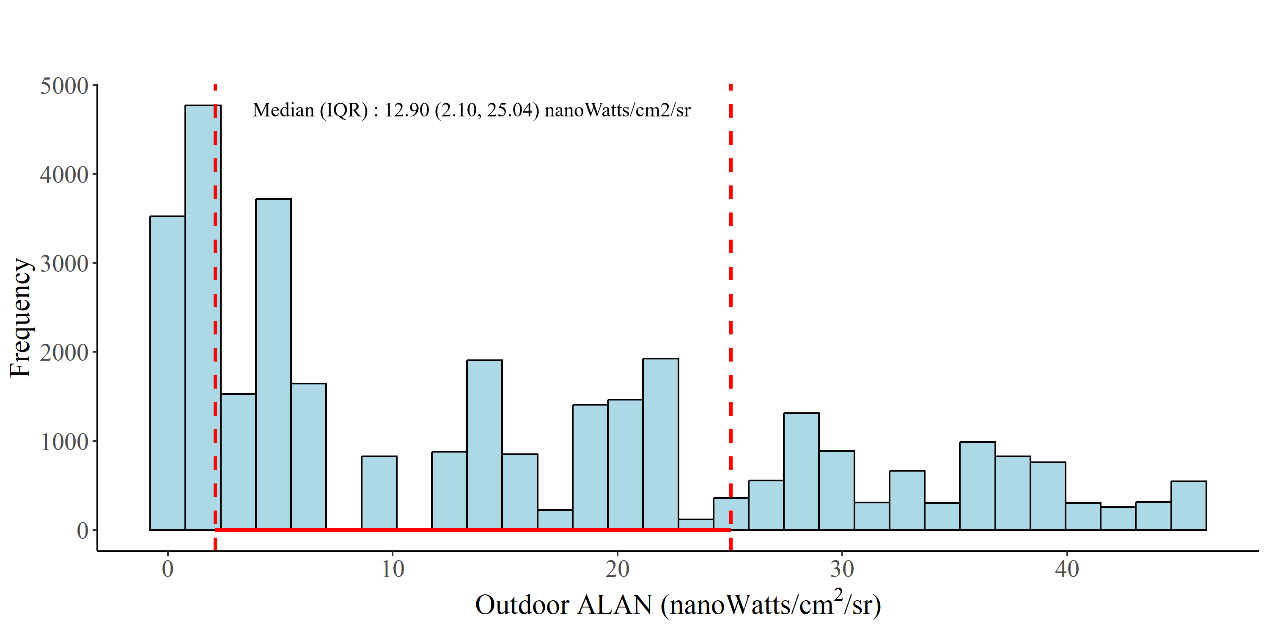
 **Fig. S3**

Histogram of outdoor artificial light at night in this study (n = 33,160).
